# Evidence of *Microdochium* Fungi Associated with Cereal Grains in Russia

**DOI:** 10.3390/microorganisms8030340

**Published:** 2020-02-28

**Authors:** Tatiana Yu. Gagkaeva, Aleksandra S. Orina, Olga P. Gavrilova, Nadezhda N. Gogina

**Affiliations:** 1 All-Russian Institute of Plant Protection (VIZR), St.-Petersburg, 196608 Pushkin, Russia; orina-alex@yandex.ru (A.S.O.); olgavrilova1@yandex.ru (O.P.G.); 2 All-Russian Scientific Research and Technological Institute of Poultry, Sergiev Posad, 141311, Moscow region, Russia; n.n.gogina@mail.ru

**Keywords:** *Microdochium nivale*, *M. majus*, *M. seminicola*, seed-borne fungi, morphology, DNA, mycotoxins

## Abstract

In total, 46 *Microdochium* strains from five different geographic regions of Russia were explored with respect to genetic diversity, morphology, and secondary metabolites. Based on the results of PCR, 59% and 28% of the strains were identified as *M. nivale* and *M. majus*, respectively. As a result of sequencing four genome regions, namely ITS, LSU, BTUB, and RPB2 (2778 bp), five genetically and phenotypically similar strains from Western Siberia were identified as *M. seminicola*, which, according to our findings, is the prevalent *Microdochium* species in this territory. This is the first record of *M. seminicola* in Russia. Attempts were made to distinguish between *Microdochium* species and to identify species-specific morphological characteristics in the anamorph and teleomorph stages and physiological properties. We examined the occurrence frequency of conidia with different numbers of septa in the strains of *Microdochium*. The predominance of three-septate macroconidia in *M. majus* was higher than that in *M. nivale* and typically exceeded 60% occurrence. Most *M. majus* and *M. nivale* strains formed walled protoperithecia on wheat stems. Only three strains of *M. majus* and one strain each of *M. nivale* and *M. seminicola* produced mature perithecia. The growth rate of *M. seminicola* strains was significantly lower on agar media at 5–25 °C than those of *M. majus* and *M. nivale* strains. Multimycotoxin analysis by HPLC-MS/MS revealed that the strains of three *Microdochium* species did not produce any toxic metabolites.

## 1. Introduction

In 2016, numerous head blights and necrotic lesions on the flag leaves of winter wheat plants were observed in the Southern European region of Russia. The study of DNA isolated from these wheat parts revealed the two pathogens responsible for these symptoms: *Microdochium nivale* (Fr.) Samuels & I.C. Hallett and *M. majus* (Wollenw.) Glynn & S.G. Edwards [1]. This situation with cereal diseases has led to a significant increase in attention to the precise identification of *Microdochium* fungi and their geographic distribution.

Information about the diseases, such as pink snow mold, grass decay, leaf spots, and head blight, caused by *Microdochium* fungi has been published previously [2,3,4,5,6,7,8]. *Microdochium* fungi were first identified in 1849 by Fries as *Lanosa nivalis*, which was later (1886) reclassified and placed in the genus *Fusarium* as *F. nivale* Ces. ex Berlese & Voglino. The recognition of two varieties, primarily based on the difference in conidial dimensions and septation, was first proposed by Wollenweber [9] as *F. nivale* var. *nivale* and *F. nivale* var. *majus*, and then by Gams and Müller [10] as *Gerlachia nivalis* var. *nivalis* (Ces. ex Sacc.) W. Gams & E. Müll. and *Gerlachia nivalis* var. *major* (Wollenw.). Later, the reclassification of *F. nivale* into *Microdochium* as *M. nivale* var. *nivale* (*Mn* var. *nivale*) and *M. nivale* var. *majus* (*Mn* var. *majus*) was formulated by Samuels and Hallett [11].

Other studies [12,13] suggested that distinct varieties did not exist because they could not discriminate fungal strains using conidial morphology, conidiogenesis, or response to fungicides. A recent examination of the elongation factor 1-α gene led to the reclassification of two varieties of *M. nivale* as separate species, *M*. *nivale* and *M. majus* [14].

The sexual morph of *Microdochium* for many years was called *Monographella* Petr. Following the implementation of “one fungus, one name” nomenclature, retaining the older name was preferred and therefore the genus name *Microdochium* was given priority [15]. In support of this opinion, we used the name *Microdochium* to describe both the anamorph and teleomorph reproductive stages of the fungi.

Microscopy does not provide further insights about the identity and species prevalence of *Microdochium* fungi on plants in different environmental conditions [16,17,18]. In this regard, our current knowledge of *Microdochium* fungi is relatively limited. A potent approach for the rapid identification of fungal specimens is DNA technology that allows the generation of novel species hypotheses and provides guidance for biodiversity and ecological studies. The diversity of *Microdochium* fungi as members of *Sordariomycetes* has recently been highlighted [15,19], where a range of species were described based on morphological and molecular approaches. Currently, this genus includes more than 40 species [20]; however, it seems that only *M. nivale* and *M. majus* are distributed over a large area. An accurate identification of *Microdochium* fungi to taxonomic rank would reveal the most common species in a habitat and estimate their biological characters, putative harmfulness, and distinctive features.

In the present study, we aimed to explore the diversity of *Microdochium* fungi collected from various geographic regions of Russia and to characterize their features.

## 2. Materials and Methods

### 2.1. Microdochium Strains

During 2016–2018, we isolated from small-grain cereals 35 strains, which, according to a visual diagnosis, were defined to belong to the genus *Microdochium* (Table 1). In addition, we examined ten strains from cereal plants during 2001–2014, which are stored as *M. nivale* in the collection of the Laboratory of Mycology and Phytopathology of the All-Russian Institute of Plant Protection. The analyzed *Microdochium* isolates originated from different geographical regions of Russia: 7 strains from the Northwestern region (Kaliningrad, Leningrad, and Pskov), 9 strains from the Central European region (Bryansk, Voronezh, and Belgorod), 13 strains from the Southern European region (Krasnodar, Stavropol, and North Ossetia), 9 strains from the Volga region (Tatarstan, Penza, and Mordovia), and 7 strains from the West Siberian region (Novosibirsk, Krasnoyarsk, Tyumen, and Altay).

In total, 38 strains were isolated from wheat (*Triticum aestivum* L.), 4 from barley (*Hordeum vulgare* L.), 2 from oat (*Avena sativa* L.) and 1 from rye (*Secale cereale* L.). Among them, 39 strains were isolated from the grain, 2 from the spikes, and 1 from the spot of a wheat flag-leaf. Additionally, four strains obtained in the spring period from plants with symptoms of snow mold were included in this study: three from wheat (the Southern European region, Belgorod) and one from rye (the Northwestern region, Leningrad).

Furthermore, one strain of *Microdochium* MFG 59862 was isolated in 2016 from the seeds of oat cv. Bai Yan 7, which were obtained from China for sowing in the nursery of the Vavilov Institute of Plant Genetic Resources (VIR) in the Northwestern region. This isolate has attracted attention, as it forms an abundant orange mass of conidia on potato sucrose agar (PSA) from the beginning of its first appearance.

For each isolate that sporulated during cultivation, a single-spore culture was obtained. For the isolates that did not sporulate under the proposed growth conditions, the cultures were derived from the tips of actively growing hyphae from the edge of the colony.

All strains are maintained in the collection of the Laboratory of Mycology and Phytopathology in the All-Russian Institute of Plant Protection.

### 2.2. DNA Extraction, PCR, and Sequencing

Initially, the fungal isolates were genetically typed by DNA-based methods. The total DNA from the mycelium of fungi cultivated on PSA was isolated using a genomic DNA purification kit (Thermo Fisher Scientific, Vilnius, Lithuania) according to the modified manufacturer’s protocol. Molecular identification was performed using the pair of variety-specific PCR primers for *Mn* var. *majus* Mnm2F/R and *Mn* var. *nivale* Y13NF/R [21,22]. All reactions were run according to the author’s instructions.

The large subunit rDNA region (LSU) of the DNA was amplified using LSU1Fd [23] and LR5 [24] primers. The primers V9G [25] and ITS4 [26] were used to amplify part (ITS) of the nuclear rDNA operon spanning the 3′ end of the 18S rRNA gene, the first internal transcribed spacer (ITS1), the 5.8S rRNA gene, and the second ITS region (ITS2). Part of the beta-tubulin gene region (BTUB) was amplified using primers Btub526F and Btub1332R [27], and for the RNA polymerase II second largest subunit gene (RPB2) primers RPB150F [27] and fRPB2-7cR [28] were used. Amplification of LSU and ITS regions was performed as described previously [29]. The conditions for BTUB and RPB2 followed Jewell and Hsiang [27]. The sequencing of the fragments was carried out on an ABI Prism 3500 sequencer (Applied Biosystems, Hitachi, Japan) using the BigDye Terminator v3.1 cycle sequencing kit (Applied Biosystems, Foster City, CA, USA). The manual editing of the nucleotide sequences, obtaining consensus sequences of each strain, and aligning were performed using the Vector NTI Advance 10 program (Thermo Fisher Scientific, Carlsbad, CA, USA) and MEGA X program https://www.megasoftware.net/ [30]. Basic Local Alignment Search Tool (BLAST) was used to perform similarity searches, by comparing the consensus sequences with other sequences in NCBI’s GenBank database https://www.ncbi.nlm.nih.gov/genbank/ to identify the closest matching sequences that were added to the alignment (Table 2).

To address the phylogenetic relationships among taxa maximum likelihood (ML), maximum parsimony (MP) analysis was conducted using the MEGA X program as well as Bayesian inference (BI) by MrBayes v. 3.2.1 on the Armadillo 1.1 platform [31]. Nodal support was assessed by bootstrap analysis on 1000 replicates.

Sequence data, alignment and phylogenetic tree were deposited in GenBank and TreeBASE (http://purl.org/phylo/treebase/phylows/study/TB2:S25464).

### 2.3. Characterization of the Anamorph

After establishing the taxonomic status of the strains, their properties and traits were compared. The morphological definition of the fungi was performed with the aid of taxonomic keys and descriptions [15,32].

The phenotypic characterization of every strain was carried out macro- and microscopically when the fungal cultures were cultivated on PSA and oatmeal agar (OA) at a temperature range of 5–30 °C for 14–30 days under different lighting conditions: in the dark, in the light, alternating lighting (16 h white fluorescent light/8 h dark), and under erythemal lamps (UF, LE-30, wavelength 280–380 nm).

The type of aerial mycelium, color, pigmentation, and growth rate of fungal colonies on PSA and OA were evaluated. Additionally, the conidial masses, shape, numbers of septa, and size of presented macroconidia or other morphologic changes in growing hyphae were analyzed on PSA. The preparations were examined with an Olympus BX53 microscope (Olympus, Tokyo, Japan) connected to a SUBRA camera (Jenoptik, Jena, Germany) using differential interference contrast (DIC) and phase contrast (PC). The mean and standard error (SE) of the length and width of more than 30 macroconidia according to the numbers of septa were measured whenever possible for the strains using PROGRES GRYPHAX software (Jenoptik, Jena, Germany).

### 2.4. Characterization of the Teleomorph

To stimulate the production of the teleomorph stage in self-mating experiments, the stem segments of mature wheat were autoclaved in glass tubes with 3 mL of 2% water agar (WA). Each wheat stem was inoculated by the mycelium of every strain separately. The tubes were incubated under alternating light (16 h white fluorescent light/8 h dark) at 24 °C. After 3 months, 2 mL of WA was added to the bottom of every tube to increase the humidity. The stems were screened every 2–3 weeks for 6 months to detect the presence of ascomata. Two experimental tubes were used for every *Microdochium* strain.

Fungal structures were removed from the stem and placed on a glass slide and submerged in a drop of distilled water containing 0.1% Tween 80 (Panreac, Spain) and methylene blue (Panreac, Spain). Sexual morphs were maintained in a water drop for 10–20 min and periodically pressed with a cover slide.

Ascomata, including asci, ascospores, and paraphyses, if present and extruded from the perithecia, were visualized and photographed under an Olympus BX53 microscope and an Olympus SZX16 stereomicroscope connected to a PROKYON camera (Jenoptik, Jena, Germany). The length and width were calculated for at least ten fungal structures.

### 2.5. Temperature response and Growth Rate

The strains of three *Microdochium* species were cultivated on PSA and OA. To compare their growth, the mycelial plugs of each strain (5-mm diameter) obtained from the colony growth on PSA were individually placed surface downward on the medium in the center of each Petri dish. The growth rate of the strains of *M. nivale* (*n* = 14), *M. majus* (*n* = 7), *M. seminicola* Hern.-Restr., Seifert, Clear & B. Dorn (*n* = 5), and *Microdochium* sp. (strain MFG 59862) cultivated on PSA in the dark at 5, 15, 20, 25 and 30 °C was determined.

The fungal strains were cultivated on OA at 20 °C under alternating lighting (16 h white fluorescent light/8 h dark). The growth rates were calculated as the mean values of the diameters of six-day-old fungal cultures, measured in two perpendicular directions around the grown colony. All measurements were made in duplicate.

### 2.6. Determination of Toxic Secondary Metabolites

HPLC-MS/MS system–tandem liquid chromatography–mass spectrometry was used for the detection of different secondary metabolites in seven selected strains of fungi: *M. majus* (MFG 60127 and 60129), *M. nivale* (MFG 59144 and 60144), *M. seminicola* (MFG 60132 and 60139), and *Microdochium* sp. (MFG 59862). One *Fusarium* *langsethiae* Torp et Nirenberg strain MFG 270611 was also included in the study as a fungal species that is a strong producer of type-A trichothecenes [33,34].

The strains were cultivated in 750-mL Erlenmeyer flasks containing 50 g of polished autoclaved rice moistened by 30 mL of water. In every flask, the rice sample was mixed with 1 mL of fungal suspension at 10^5^ CFU/mL; in the control, an equivalent volume of sterile water was added. All flasks were stored at 20 °C for two weeks in a MIR-154-PE incubator (Sanyo, Osaka, Japan). To prevent rice from packing in clumps, the substrate was loosened by shaking the flask daily.

The samples of rice with fungal cultures were dried at 55 °C for one day. Then, 20 g grain samples were homogenized separately in sterilized grinding chambers of a batch mill (Tube Mill control, IKA, Königswinter, Germany).

Metabolites from every 5 g of rice flour sample were extracted with 20 mL of extraction solvent (acetonitrile/water/acetic acid, 79:20:1, v/v/v) for 90 min using a Biosan PSU-20i rotary shaker (Biosan, Latvia). The detection and quantification were performed on an AB SCIEX Triple Quad ™ 5500 MS/MS system (Applied Biosystems, Foster City, US) equipped with a TurboV electrospray ionization (ESI) source and a 1290 series Agilent Infinity UHPLC system (Agilent Technologies, Waldbronn, Germany). The chromatographic separation was performed at 25 C on a Gemini^®^ C18 column, 150 × 4.6 mm i.d. (Phenomenex, Torrance, CA, USA). The analysis of the mycotoxins was carried out as described previously [35,36]. In total, 31 mycotoxins were analyzed: 3-acetyldeoxynivalenol, 15-acetyldeoxynivalenol, aflatoxin B1, aflatoxin G1, alternariol, alternariol monomethyl ether, beauvericin, cyclopiazonic acid, deoxynivalenol, deoxynivalenol-3-glucoside, diacetoxyscirpenol, fumonisin B1, B2, and B3, HT-2 toxin, fusarenon-X, moniliformin, mycophenolic acid, neosolaniol, nivalenol, ochratoxin A and B, patulin, sterigmatocystin, tentoxin, tenuazonic acid, T-2 toxin, T-2 triol, zearalenone, α-zearalenol, and β-zearalenol.

### 2.7. Statistical Analysis

Statistical analyses were performed using Microsoft Office Excel 2007 and Statistica 10.0 (ANOVA) programs. The distributions were verified to be approximately normal for all parametric models used. A standard t-test was used to determine statistically significant differences between the means. The results were considered to be significant when *p* < 0.05.

## 3. Results

The strains analyzed in this study were selected without any previous observations of the morphological details of different microstructures and only by their superficial resemblance to *Microdochium* fungi. Since the formation of clear morphological traits took time, we used DNA identification first. Then, the morphological and other characters were analyzed in accordance with the established species taxa.

### 3.1. Molecular Identification of Strains

**Figure 1:**
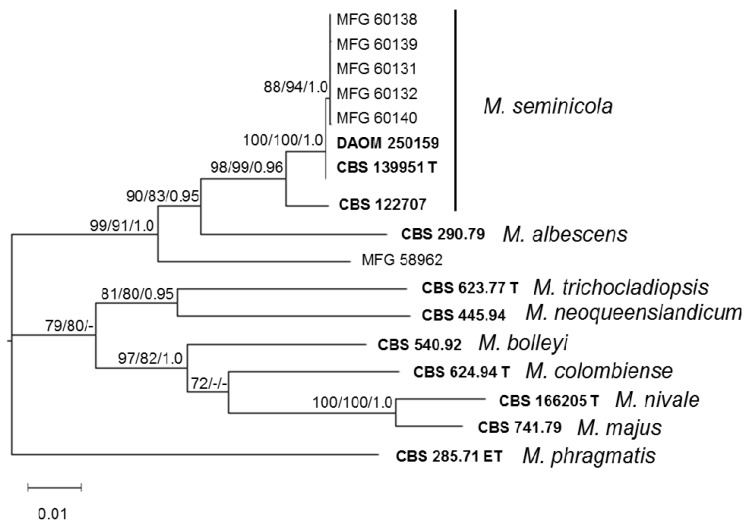
Maximum likelihood (ML) phylogenetic tree based on DNA sequence data from four loci (ITS, LSU, BTUB, and RPB2) of *Microdochium* species. ML and maximum parsimony (MP) bootstrap support values > 70%, followed by Bayesian posterior probability scores > 0.95 are shown at the nodes. The tree was rooted with *Microdochium phragmitis*. T, ex-type strain; ET, ex-epitype strain.

Specific PCR primer sets [21,22,37] were used to differentiate between *Microdochium* species. Among the 46 analyzed strains, 27 were *M. nivale* and 13 were *M. majus* based on the PCR results. Six DNA samples of the strains were indistinct in the amplification results for unclear reasons. Optimization attempts did not improve the situation, and clear products were not obtained for these DNA samples with the primer sets. Among these DNA samples, five strains were obtained from spring wheat grains cultivated in West Siberia, and one strain, MFG 58962, was isolated from the Chinese oat grain. The identification of strains by sequencing was necessary to compensate for the failure of PCR with the specific primers. The analysis of combined ITS, LSU, BTUB, and RPB2 (2778 bp) sequences alignment confirmed that the five strains from the West Siberian region belong to *M. seminicola* species. If all three consensus tree topologies place the single strain from China MFG 59862 outside of *M. seminicola* and *M. albescens,* it might represent a new taxon related to these two species, as it is clearly part of the clade that contains these two species (Figure 1).

The molecular identification of *Microdochium* strains from the Northwestern region of Russia revealed that 5 strains were *M. nivale* and *2* strains were *M. majus* (Supplementary Materials Figure S1A). While the majority of the strains from the Central European region were identified as *M. nivale*, the proportions of *M. majus* and *M. nivale* were approximately equal in the Southern European region. All strains from the Volga region were characterized as *M. nivale*. In the West Siberian region, two strains belonged to *M. majus* and *M. nivale*, and, surprisingly, the others were *M. seminicola.*

All four strains isolated from the leaves with symptoms of snow mold were identified as *M. nivale.*

### 3.2. Characteristics of the Anamorph

**Figure 2:**
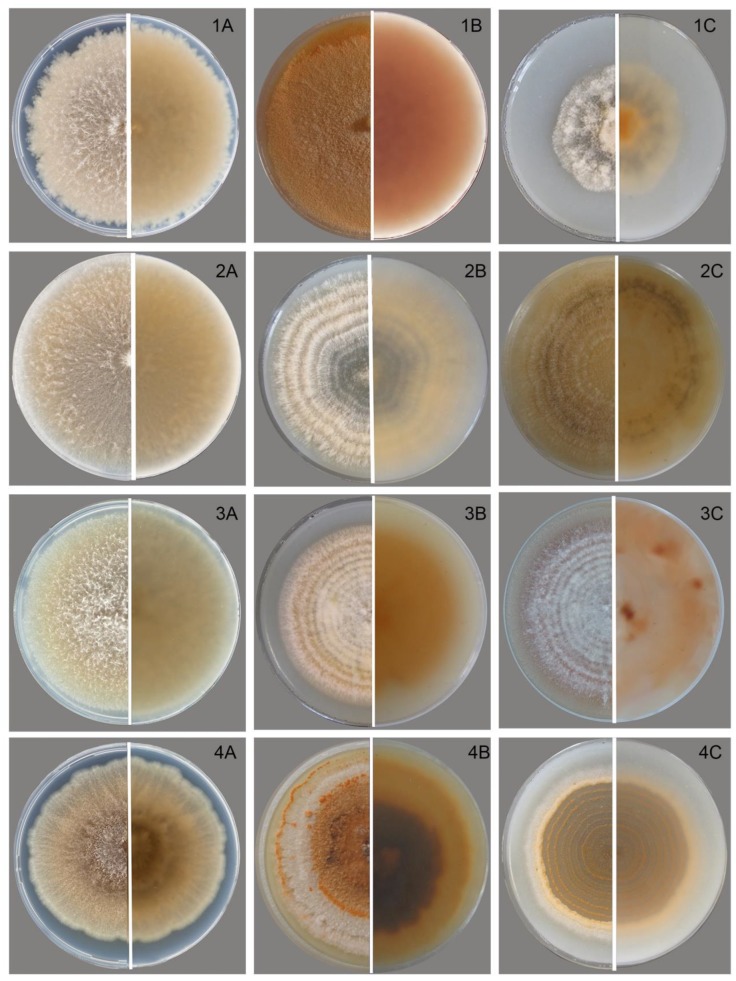
Cultures of *Microdochium* fungi in a 80 mm Petri dishes at 25 °C. *M. majus*: (1**A**) on PSA in dark, two weeks; (1**B**) on PSA under UF, two weeks; (1**C**) and on OA, 16/8 light and dark, one week. *M. nivale*: (2**A**) on PSA in dark, two weeks; (2**B**) on OA 16/8 light and dark, one week; (2**C**) and on OA, 16/8 light and dark, four weeks. *M. seminicola*: (3**A**) strain on PSA in dark, two weeks; (3**B**) on OA, 16/8 light and dark, one week; (3**C**) and on OA, 16/8 light and dark, two weeks. *Microdochium* sp. strain MFG58962: (4**A**) on PSA in dark, two weeks; (4**B**) on PSA, light, four weeks; and (4**C**) on OA, 16/8 light and dark, one week.

The strains of *M. majus* and *M. nivale* on PSA and OA in the dark formed colonies with cobweb-like, loose to fasciculate, densely floccose, and not abundant aerial mycelium (Figure 2). Typically, the *M. nivale* strains characterized by aerial mycelium were more cobweb-like and spread over the surface agar than the *M. majus* and *M. seminicola* strains. Under alternating lighting, the tested strains formed colonies with concentric rings of thin, dense mycelium growth.

On all media, when the fungi were cultivated in the dark, the color of the colonies was whitish-cream. Under light, the aerial mycelium turned a peach color. The reverse side was not pigmented, reflecting the state of the colony and the aerial mycelium. The cultivation of strains under UV light caused the colonies to acquire a bright salmon color.

At the beginning of growth, the colonies of all studied *Microdochium* strains were similar, except for *Microdochium* sp. strain MFG 58962 from China, which revealed intense conidial production on all media after seven days of cultivation. The slimy sporodochia (often appearing as a pionnotal layer) with profuse conidial masses appeared on all media, which was a remarkable feature of this *Microdochium* sp. strain MFG 58962.

After two weeks, in some strains of *M. majus* and *M. nivale*, a few aerial conidia were observed in the mycelium that were usually unicellular, single-septum, or multicellular, occasionally up to three-septate. Later, depending on the strain, apricot-brown sporodochia masses of macroconidia on single or branched conidiophores ending with annellated conidiogenous cells formed.

**Figure 3:**
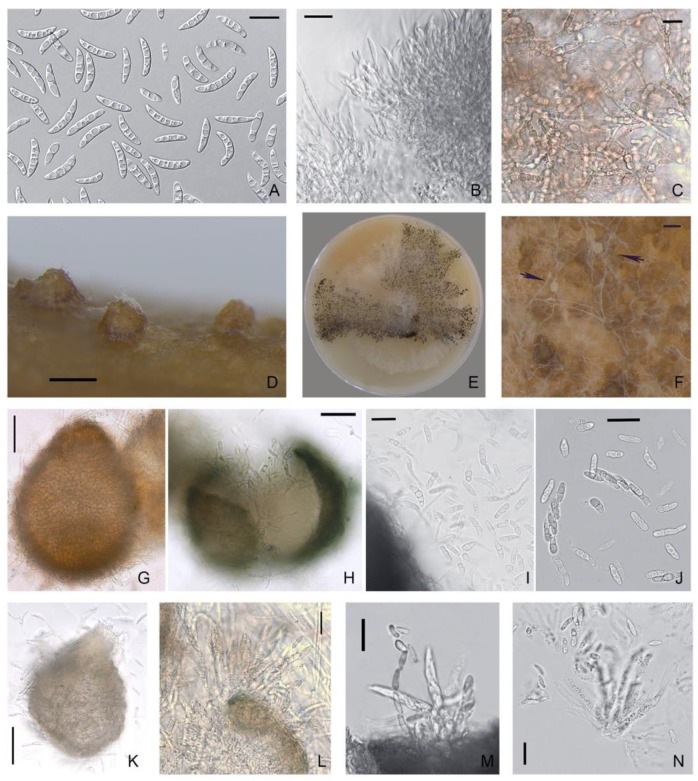
*M. majus*: (**A**) conidia; (**B**) aggregated conidiophores with conidiogenous cells; (**C**) dark brown hyphal cells; (**D**,**E**) ascomata; (**F**) ascogenous hyphae; (**G**) intact protoperithecium; (**H**) ascospores exuded from crashed perithecium; (**I**,**J**) ascospores; (**K**) ascospores exuded from the ostiole; (**L**,**M**) prototunicate asci with thickened annulus; and (**N**) asci, ascospores, paraphyses. Media: (**A**–**C**,**E**–**K**) PSA; **(D**,**L**–**N**) wheat stems. Scale bars: (**A**–**C**,**I**,**J**,**L**,**M**,**N**) 20 μm; (**G**,**H**,**K**) 50 μm; (**D**,**F**) 100 μm.

**Figure 4:**
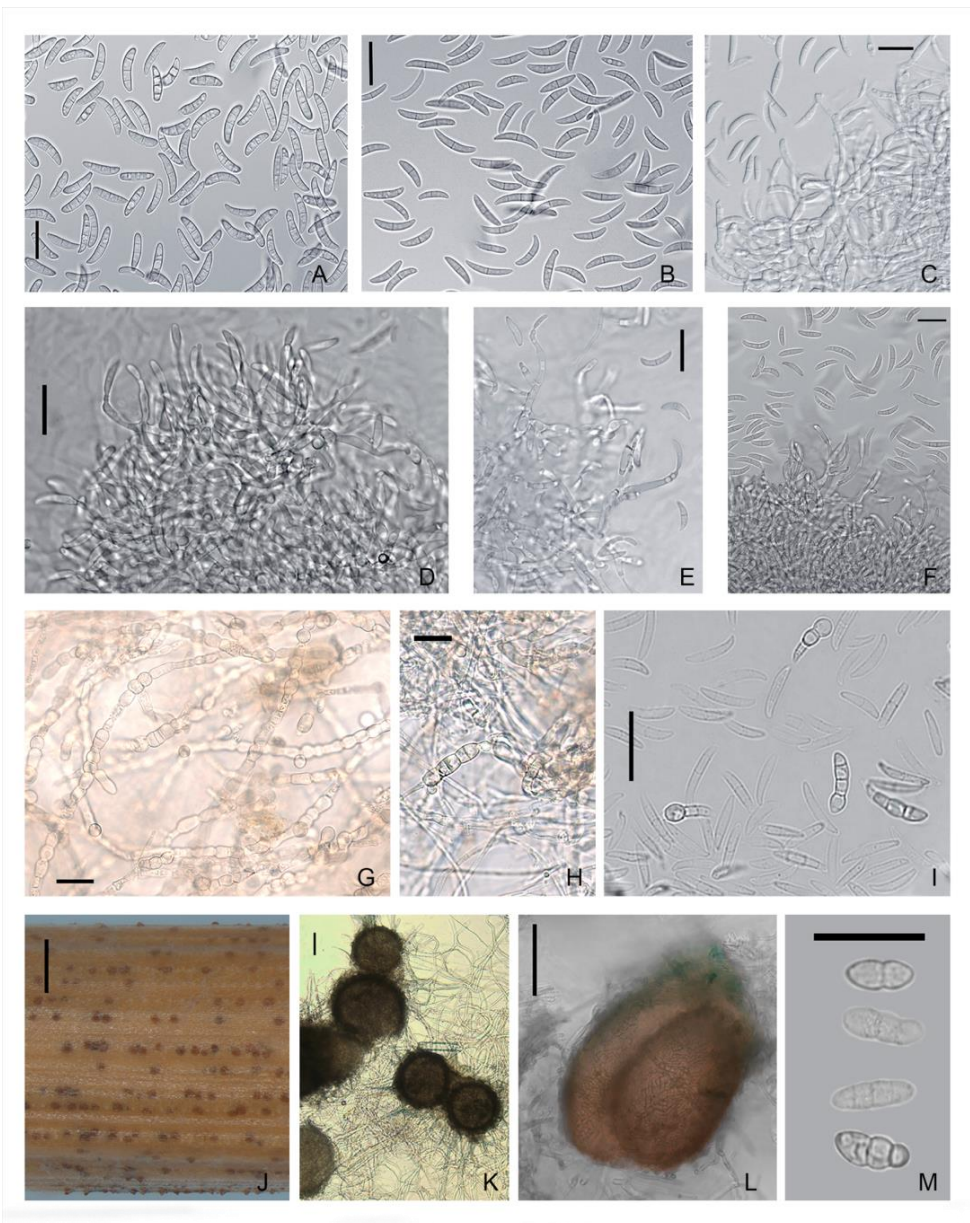
*M. nivale*: (**A**,**B**) conidia; (**C**–**F**) aggregated conidiophores with conidiogenous cells; (**G**,**H**) dark brown hyphal cells; (**I**) thick walled conidial cells; (**J**–**L**) ascomata; and (**M**) ascospores. Media: (**A**–**I**,**L**,**M**) PSA; (**J**,**K**) wheat stems. Scale bars: (**A–I**,**M**) 20 μm; (**J**) 1 mm; (**K**,**L**) 50 μm.

The conidiophores of *M. majus* and *M. nivale* arose laterally on hyphae as single annelides or were sparsely branched and aggregated; these hyphae elongated up to 50 μm with age (Figure 3 and Figure 4). The annelides were subcylindrical with a percurrently elongating apical zone with a size of 9.5 × 3.5 (16–4.8 × 2.7–5.8) μm.

In *M. majus* and *M. nivale*, the sporodochial macroconidia on PSA were mostly single-septum or three-septate (rarely zero-, two-, four- or five-septate), fusiform and straight to slightly curved; the apical cells were gradually tapering and rounded, and flattened at the base. The maximal sizes of 4–5-septate conidia in both *Microdochium* species reached 32.6–34.5 × 4.5–5.7 μm. The strains of *M. majus* typically formed a few pear-shaped single-celled or 1–2-septate conidia (8.5–14.2 × 5.3–6.3 μm).

**Figure 5:**
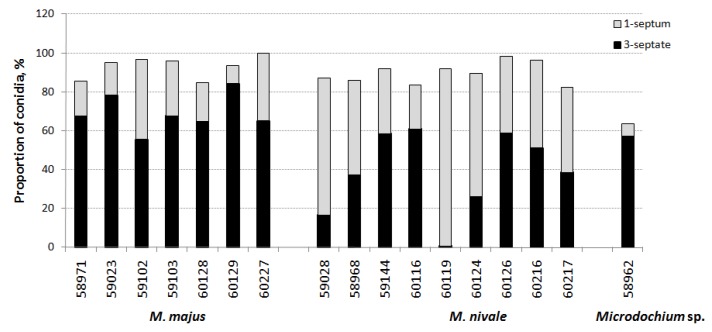
Proportion of macroconidia with one septum and three septa in *Microdochium* fungi on PSA.

The frequencies of occurrence of single-septum or three-septate conidia in the strains of these two species were assessed. According to the calculations, the share of single-septum conidia ranged from 9.5 to 40.9%, and that of three-septate conidia was 55.5–84.2% among all macroconidia in the *M. majus* strains (Figure 5). In *M. nivale*, these measurements were 22.7–90.1% and 0.6–61.0%, respectively. On average, the occurrence of three-septate macroconidia in *M. majus* was 68.9 ± 9.4%, while that in *M. nivale* was 38.7 ± 21.0%.

Based on the three-septate conidial occurrence, *M. nivale* and *M. majus* differed significantly (*p* = 0.13). However, the dominant macroconidia in some strains overlapped for individual species. In some strains of *M. nivale*, the proportions of conidia with three septa exceeded 50% of the detection frequency, and, therefore, they were similar to *M. majus* in the visual analysis. Single-septum conidia were low in frequency in *M. nivale* strains MFG 59144, 60116, 60126.

**Figure 6:**
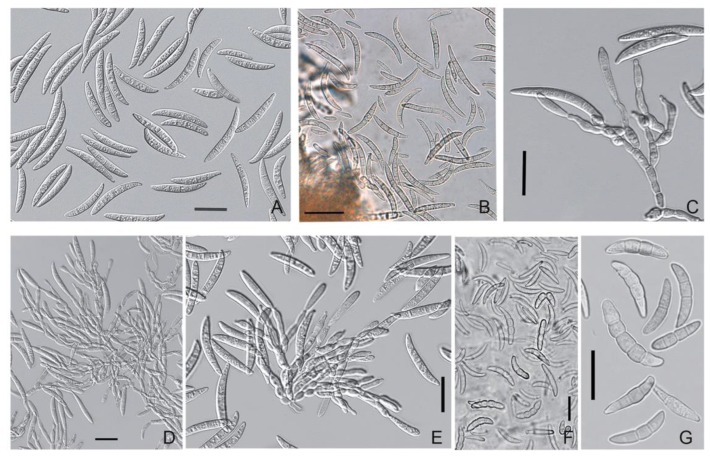
*Microdochium* sp. strain MFG 58962: (**A**,**B**) conidia; (**C**–**E**) conidiophores with conidiogenous cells; and (**F**,**G**) thick walled conidial cells. Media: (**A**–**E**) PSA; (**F**,**G**) oatmeal agar. Scale bars: (**A**–**G**) 20 μm.

The conidiophores of *Microdochium* sp. strain MFG 58962 arose laterally as single annelides or were sparsely branched on hyphae that elongated with age (up to 60 μm). Annelides were subcylindrical with a percurrently elongating apical zone of 10.7 × 4.2 (8–13 × 2.8–5.8) μm. The *Microdochium* sp. strain produced conidia cylindrical to fusiform, straight, often with a curved upper part, hooked and slightly constricted apical cells and flattened basal cells (Figure 6). Approximately 57% conidia were three-septate (0–7) with a size of 28.6 × 4.6 (19.2–40.5 × 3.25–5.5) μm. Additionally, this strain of *Microdochium* sp. strain MFG 58962 had a high proportion of five-septate macroconidia (18.7%) with a size of 37.6 × 4.7 (30.3–44.8 × 3.8–5.5) μm.

**Figure 7:**
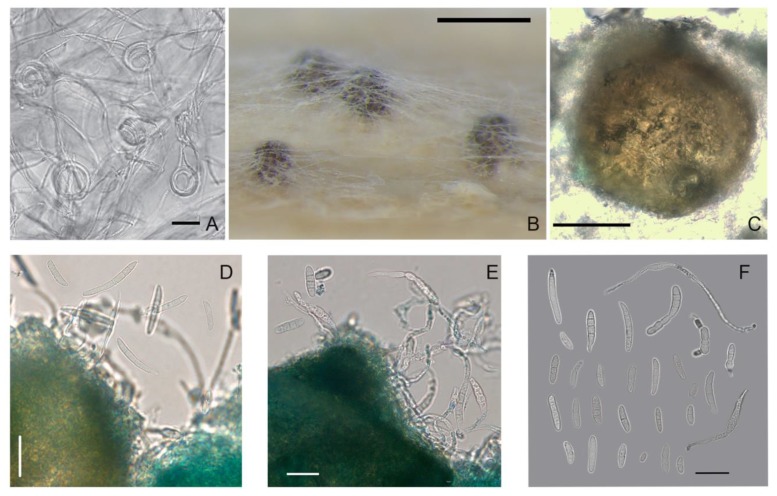
*M. seminicola* strain MFG 60138: (**A**) the coils in hyphae; (**B**,**C**) ascomata on wheat stems; (**D**) conidia; (**E**) germinated ascospores that exuded from perithecium; and (**F**) variety of formed spores, some of them germinated. Media: (**A**) PSA; (B–F) wheat stems. Scale bars: (**A**,**D**–**F**) 20 μm; (**B**) 200 μm; (**C**) 50 μm.

Only one West Siberian strain, *M. seminicola* MFG 60138 (Novosibirsk), produced a few conidia, but it occurred after the experiment estimating the growth rate at 25 °C on PSA, after the *Microdochium* cultures has been kept for approximately one month at 5 °C. The same strain produced a few conidia in the experiment on the production of perithecia on the wheat stem segments after one month of cultivation (Figure 7). The shapes of these a few conidia and the sizes of the three-septate 30.6 × 4.0 (25.5–33.5 × 3.6–4.6) μm and single-septum 13.4 × 3.4 μm conidia were similar to those of *Microdochium* sp. MFG 58962. The other *M. seminicola* strains that originated from West Siberia remained sterile and could not sporulate on any media or under different living conditions (which included many more variants than described in this publication).

The average length of conidia with three septa was 22.9 ± 1.6 μm in *M. majus*, 19.6 ± 2.7 μm in *M. nivale*, and 28.7±0.6 μm in *Microdochium* sp. strain MFG 58962 (Supplementary Materials Figure S1B), which differed significantly (p = 0.004–0.02). The average width of three-septate conidia was 5.0 ± 0.5 μm in *M. majus*, 3.5 ± 0.7 μm in *M. nivale*, and 4.6 ± 0.05 μm in *Microdochium* sp. strain MFG 58962, exhibiting a significant difference (p = 0.01–0.02).

In *M. majus* and *M. nivale* strains and *Microdochium* sp. strain MFG 58962, dark brown hyphae cells were observed after cultivation for a few weeks on agar media. These melanized intercells of the hyphae of the substrate mycelium were visible under the aerial mycelium and on the reverse side of the Petri dish. The chlamydospore-like structures, as thick-walled cells that were darker than the rest of the cell, were also detected in the conidia.

The hyphae of *M. seminicola* strains on agar media frequently formed coils that were 22.8 × 24.7 (18.6–29.6) μm.

### 3.3. Characteristics of the Teleomorph

In the self-mating experiments, the first visible superficial fruiting bodies were formed in vitro, but not matured, on the stems of wheat in one month. After 4–6 months of the experiment, protoperithecial structures, such as young-walled immature perithecia before ascus formation [38], were noted in 5/13 *M. majus* strains, 6/27 *M. nivale* strains, 2/5 *M. seminicola* strains, and in *Microdochium* sp. strain MFG 58962. All other strains did not form ascomata on the stems of wheat.

However, during the cultivation of *Microdochium* strains on nutrient agar, some strains also formed ascomata on the surface of the medium, which typically formed gregariously in small groups. Thus, in the period from two weeks to two months, the maturing perithecia were fixed on PSA in three strains of *M. majus* (MFG 60127, 60128, and 59102) and one strain of *M. nivale* (MFG 60216). In other *Microdochium* strains, only protoperithecia were detected, of the size as the dark brown and black bodies, ranging 100–245 × 100–260 μm.

The formation of sexual morphs in *Microdochium* strains proceeded gradually in several steps beginning with the looping of a hyphal filament and the generation of small, light brown protoperithecia with heavily pigmented and densely packed fruiting bodies. These were immersed and subepidermal, of different sizes and shapes, such as ovoid to pyriform with a short, ostiolate neck, nonstromatic, solitary or less commonly in a pair, and smooth or minutely verrucose. The texture of the wall was rough, textura angularis-epidermoidea, and of variable color, i.e., initially light brown and becoming blackish to brown with age.

The dimensions of the black-brown ascomata on wheat stems were in the range of 100–170 × 90–160 μm. A few fertile perithecia on wheat stems were detected only in one strain of *M. majus* (MFG 60127) and one strain of *M. seminicola* (MFG 60138) after 3–4 months of cultivation (Table 3).

The examination of the fruiting bodies under the microscope revealed single ascospores that exuded from the ostiole or the wall break of the mature perithecium. This is typical for prototunicate asci that have no active shooting mechanism. The thin delicate wall of the ascus at maturity decayed or dissolved in water, thereby releasing the ascospores. These ascospores germinated rapidly, and they had already germinated when emerging from the crushed perithecia.

Obvious prototunicate asci with thickened annuli and thin-walled paraphyses were observed in *M. majus* MFG 59102 on PSA. The dimensions of the mature asci in *M. majus* were 45–60 × 6.0–8.0 μm, and they were oblong, clavate and straight with eight ascospores. The ascospores were hyaline, oblong, straight, or slightly curved, single-septum or three-septate and often narrowed at the septum. Typically, the size of the single-septum ascospores ranged 9.5–19 × 2.8–6.0 μm and that of the three-septate ascospores ranged 12.3–23.6 × 3.0–6.2 μm.

In *M. nivale* strain MFG 60216, a few single-septum and three-septate ascospores were detected. Considering that the number of ascospores in the *M. nivale* was small, the differences between their sizes and those of the abundantly presented ascospores in the *M. majus* strains could not be evaluated. Ascospores in asci with one and three partitions could coexist, but three-septate ascospores were more common. The single ascospore subcultures on PSA formed colonies typical of the *Microdochium* fungi.

The strains of *M. majus* and *M. nivale* maintained in the culture collection for more than five years did not form any fertile structures; they formed only conidia.

### 3.4. Temperature Response and Growth rate

**Figure 8:**
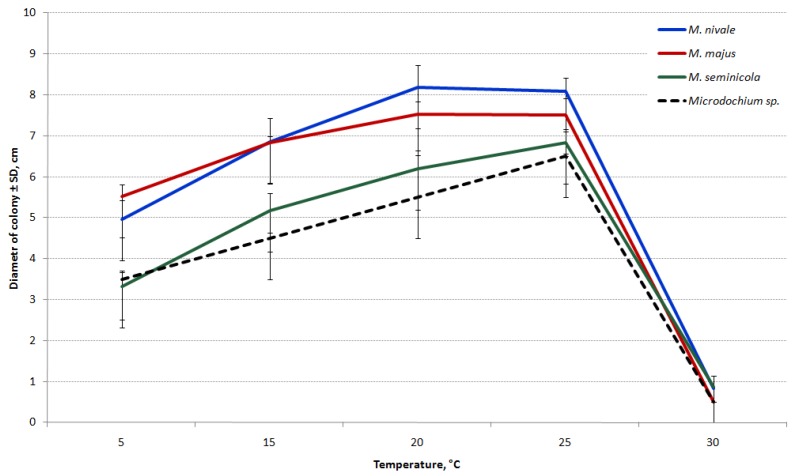
Growth curves of *Microdochium* fungi on PSA after six days of incubation at different temperatures.

The differences in the growth rates on PSA between *Microdochium* species are shown in Figure 8. While all the tested *Microdochium* strains grew at 5 °C, they completely ceased to grow at 30 °C.

The growth rates of *M. majus* and *M. nivale* strains were similar at all temperatures analyzed. The highest estimated average growth rate of these species was detected at 20–25 °C (1.17 cm/day for *M. majus* and 1.27 cm/day for *M. nivale*).

The *M. seminicola* and *Microdochium* sp. strains were relatively slow-growing and have not demonstrated the significant difference at all temperatures. The temperature for the optimal growth of these strains was 25 °C (1.14 cm/day and 0.08 cm/day, respectively). The results indicate substantial growth differences between the strains of *M. seminicola* and other *Microdochium* species within 5–25 °C.

When the strains were cultured on PSA at 20 °C, the difference in the average growth rates of *M. majus* and *M. nivale* was nearly significant. Therefore, the strains were repeatedly cultivated at this temperature on OA. Consequently, the growth rates of strains under these conditions were significantly lower than those on PSA. The mean growth rates of both *M. nivale* and *M. seminicola* were 1.0 cm/day and *M. majus* was 0.88 cm/day. As a result, substantial differences were observed in the growth rate between the strains of *M. majus* and the first two analyzed *Microdochium* species.

### 3.5. Determination of Toxic Secondary Metabolites

After inoculation, the rice grains turned a bright peach-salmon color. The sporulation of fungi did not occur on the rice grain substrate, except in the *Microdochium* sp. strain MFG 58962.

In the original autoclaved rice, tenuazonic acid was present at 64±16 ppb, and traces (≤7 ppb) of T-2 toxin, HT- 2 toxin, T-2 triol, deoxynivalenol, zearalenone, nivalenol, and tentoxin were detected. In the grain substrates inoculated with *Microdochium* strains, the analyzed metabolites were not detected in concentrations exceeding the background level.

Conversely, in the rice sample inoculated with *F. langsethiae* strain MFG 270611, trichothecene mycotoxins were found (ppb): diacetoxyscirpenol, 29.7; HT-2 toxin, 7306.0; neosolaniol, 763.4; T-2 toxin, 1660.0; and T-2 triol, 935.5.

## 4. Discussion

The *Microdochium* strains were accumulated for 18 years in our culture collection and were obtained as the result of monitoring seed-borne fungi in the different regions of Russia. The selected strains for this study were randomly taken from a large number of stored *Microdochium* strains. The results of morphology-based research were inconsistent, and distinct differences between the strains were not detected. One *Microdochium* strain, MFG 58962, attracted attention for its dark-colored culture and rapid formation of bright orange sporodochia.

The amplification of the DNA of the 46 chosen strains [21,22,37] revealed that 58.7% of the strains were *M.* *nivale* and 28.3% were *M. majus*. The remaining five strains that phenotypically resembled *Microdochium* fungi were identified as *M. seminicola* by sequencing the ITS, LSU, BTUB, and RPB2 regions of the DNA. These regions were sufficiently informative and are recommended for the differentiation of *Microdochium* species [15,39]. The phylogenetic analysis based on combined ITS, LSU, BTUB, and RPB2 sequences (2778 bp) of six *Microdochium* strains resulted in the differentiation of five of them to unambiguous monophyletic group with the *M. seminicola* strains DAOM 250159 and CBS 139951 T (Switzerland, maize).

Briefly, *M. majus* and *M. nivale* are the predominant *Microdochium* species in the European part of Russia. All *Microdochium* strains originating from the Southern European region were equally represented by *M. majus* and *M. nivale*. This territory has rarely suffered from snow mold disease because of the poor snow cover in the fields in winter and the rapid arrival of spring [40]. Recently, *Microdochium* fungi were detected on the heads, which resembled the symptoms of Fusarium head blight, in seeds, and on the flag leaves of winter wheat [1,36,41] (Supplementary Materials Figure S2).

In the Central and Northwestern European regions, *M. nivale* is predominant and accounts for 64.7% of all the analyzed strains from this territory.

In Siberia, the snow mold caused by *Microdochium* fungi has never been observed during research expeditions [42,43]. In this area, winter crops are limited, but there has recently been a tendency to increase the area under winter cereals, which can lead to the increased incidence of snow mold in cereal fields. Previously, *Microdochium* fungi were detected sporadically as seed-borne pathogens (referred to as *F. nivale*) [44].

*M. seminicola* was not described previously in Russia. Of the seven analyzed strains obtained from spring wheat grains harvested in the West Siberian region, five strains were identified as *M. seminicola*. Thus, we suggest that, in the territory of West Siberia, at least three *Microdochium* spp. exist. Obviously, *M. seminicola* causes symptomless endophytic infection in cereals, and, from an ecological perspective, additional information about the distribution of this species is essential.

Previously, *M. seminicola* strains were identified in cereal grains grown in Switzerland and Canada [15]. It was reported that, in Canadian seed testing laboratories, the sporadic occurrence of fast-growing *Microdochium* strains that often remain sterile on PDA was 3–4%. These findings are similar to our observations for *M. seminicola* in the analyzed grain seeds from the West Siberian region. It was also noted [15] that a few orange sporodochia were produced by these strains, but the sporulation disappeared after one or two transfers, resulting in sterile, relatively fast-growing light-orange colonies. The strains of *M. seminicola* from West Siberia were sterile from the beginning of the first round of isolation from the grain.

Furthermore, in our opinion, the Canadian isolate NRRL3289, which was originally deposited by W.L. Cordon in 1967 and was an exception among the carefully examined strains of *M. majus* and *M. nivale* [14], is similar to our strains of *M. seminicola.* This NRRL3289 strain was examined by Logrieco et al. [45] and described as being in a degenerated state due to multiple transfers. However, it still produced macroconidia because the studies had a single-conidium culture, which was identified as similar to *Mn* var. *majus*.

The solitary strain of *Microdochium* sp. MFG 58962 was isolated from a sample of Chinese oat seeds sown in a VIR nursery. The search for additional *Microdochium* strains in the freshly harvested oat grain from this nursery was unsuccessful (unpublished results), possibly because the identified strain was nonpathogenic and it could not adapt fast enough to the habitat.

The strain MFG 58962 initially tended to acquire the pionnotal type but also began to produce a small number of conidia with age. The morphology characteristics of *Microdochium* sp. strain MFG 58962 from China were different from other *Microdochium* species reported previously and therefore it is proposed as an unknown species. In addition, the morphological taxonomy was confirmed by the multilocus molecular phylogenetic analysis.

We have searched for further information regarding *Microdochium* fungi detected in China. In wheat and barley grains collected in different regions of China during the period 1975–1985, *M. nivale* sensu lato was detected [46]. In 2009, in Hubei, *M. nivale* var. *majus* was found in wheat grains [47]. Recently, information was published about *M. majus*, which caused a serious basal stem rot disease of wheat in Anhui province [48]. In addition, new species of *Microdochium* fungi have been described recently: *M. paspali* causing seashore paspalum disease in southern China [49] and *M. poae* causing leaf blight disease of turfgrasses in northern China [50]. We were unable to find information about *Microdochium* fungi closely related to our strain, both morphologically and according the genomic data accumulated by other studies.

The present study aimed to determine whether the strains of *Microdochium* fungi could be identified based on their morphology or to identify conditions under which they would differ significantly. This would allow an additional routine characterization method for *Microdochium* strains without molecular studies. The current results were consistent with previous data [5,13,14,16] and revealed that the accuracy of the traditional morphological identification of *Microdochium* strains was low. In the *M. seminicola* strains, the formation of conidia as the diagnostic characteristic is unreliable or difficult to observe. In visual testing, the difference between species is not distinct but is statistically significant with respect to some measurable parameters. For example, the growth rate of *M. seminicola* strains was significantly lower at 5–25 °C on the agar media compared to the growth rate of the two other closely related species.

The most evident morphological differences that readily separate *Microdochium* species are based on the number of septa and the dimensions of their conidia. According to Gerlach and Nirenberg [32], the conidia of *M. majus* (referred to as *F. nivale* var. *majus*) were larger, with lengths of 15–33 µm and widths of 4.2–6.0 µm, than those of *M. nivale* (referred to as *F. nivale* var. *majus*), with lengths of 8–27 µm and widths of <3.8 µm, and possessed a larger number of septa (1–7 compared to 0–3). Lees et al. (1995) maintained that conidial width could distinguish narrower *Mn* var. *nivale* from *Mn* var. *majus*. However, some studies questioned the validity of these morphological characteristics for taxonomic purposes [12,13].

We examined the strains to determine the septation frequency. The predominance of three-septate macroconidia in *M. majus* was higher than that in *M. nivale* and typically exceeded 60% occurrence. However, 28% of the *M. majus* strains were excluded from this observation, and the proportion of three-septate conidia was <50%. Simultaneously, for the third part of the *M. nivale* strains, this value was very close to 50–60% of conidia, which made it difficult to clearly identify the strains to species when examined under a microscope. A greater variation was noted in conidia sizes within *M. nivale* compared to other species. The length of three-septate conidia in *Microdochium* sp. strain MFG 58962 from China was longer, with a curved upper part, in comparison to those of the two other species.

Several studies mentioned that chlamydospores are not observed in *F. nivale* sensu lato [32,51]. Hernández-Restrepo et al. [15] stated that the presence of chlamydospores was “not reported” in *M. majus* and *M. nivale* and was “not observed” in *M. seminicola*. Overall, the chlamydospores in the *Microdochium* genus were assumed and described as terminal or intercalary, solitary, in chains or grouped in clusters and brown in color [15]. However, according to the information from Maharachchikumbura et al. [52] about *Microdochiaceae,* the absence of chlamydospores clearly distinguishes the fungi in the *Microdochium* genus from closely related fungi in the *Idriella* genus.

The strains of *M. majus*, *M. nivale*, and notably *Microdochium* sp. strain MFG 58962 had melanized hyphal and conidial cells with thick walls, which were similar to the chlamydospores. The manifestations of this trait are strain-dependent and observed with age independent of the temperature or media cultivation.

The *M. seminicola* strains were not identified to the species level because their cultures did not have distinct morphological differences and were commonly sterile. Nonetheless, *M. majus* and *M. nivale* strains might also need prolonged cultivation to initiate sporulation. As a result, the visual observation of new *Microdochium* strains did not conclusively identify the species, and, in most cases, we can only be sure about the genus and not the species.

In the study, most *Microdochium* strains formed abundant walled protoperithecia. Only three single-spore strains of *M. majus* and one each of *M. nivale* and *M. seminicola* produced mature perithecia with ascospores *in vitro*, suggesting the potential of these species to reproduce homothallically.

Consistent with previous findings [13,16,18,53], both *M. majus* and *M. nivale* species produced perithecia as in the current study, and *M. majus* produced perithecia in vitro more often than *M. nivale*. A low degree of variability was observed within the group of *Mn* var. *majus* compared to the *Mn* var. *nivale* group of British isolates originating from wheat [16], indicating that the first group generally reproduces homothallically, whereas the high degree of variability within the second group suggested that heterothallic reproduction was rather common. In a study [54] of *M. nivale* sensu lato isolated from grass and cereals in Canada, most of the strains did not produce perithecia. Several of the cereal strains formed these structures, some more frequently than others, but most of the perithecia were not mature.

Intriguingly, the mating type (MAT) genes and reproductive strategy in *Microdochium* fungi have not yet been completely elucidated; hence, the structure of the mating type locus and its surrounding regions has been under intensive focus [53,55]. To identify MAT genes within *Microdochium* spp. was not the objective of our current study, but it would benefit from further research to clarify the fungal sexuality of *Microdochium* spp., including the strains that we studied.

Protoperithecia formation is an endogenous property and does not require any inductive signal from the opposite mating type [56]. In our strains, protoperithecia occurred abundantly in the single spore cultures on wheat stem segments and on agar media. Fertility can be impaired in a multitude of ways by not only genetic but also environmental factors, such as temperature, light, humidity, and substrate, as well as physical factors that might affect the various developmental stages or the function of perithecia [57,58]. The laboratory conditions that we proposed for our strains may not have been suitable for the maturation of protoperithecia, and external stimuli favorable for the formation of ascospores should be identified.

Since the ascomata of *Microdochium* spp. form at different time points, and their maturation was uneven, various sizes and colors were noted by microscopy. Herein, only the mature, dark fruit bodies of *M. majus* and *M. nivale* strains forming ascospores were measured. Because of the large variations in size, no significant difference was detected between the species. However, according to our measurements, the average perithecia sizes were 143–165 × 113–163 μm; the maximally detected size reached 252 μm, which was much smaller than usually indicated for these species. For example, according to Hayashi (2016), the perithecia size of *M. majus* is 258.2 (144–334) × 219.3 (134–268) µm and that of *M. nivale* is 183.2 (152–220) × 164.8 (124–210) µm, with clear differences between the two species cultured on wheat straw segments. Hernández-Restrepo et al. [15] also reported large perithecia sizes for *M. majus* and *M. nivale* of 300 × 170 µm.

The sizes of the ascospores corresponded to those indicated by other authors and were typically shorter and thinner than the conidia [51]. In our experiments, the observed single-septum and three-septate ascospores were 9.5–23.6 µm in length and 2.8–6.2 µm in width. However, the largest sizes might be due to the swollen conidia emerging from the perithecia in water during microscopy, ready for germination. The average size of the ascospores did not differ between the strains. Nonetheless, we have not examined a sufficient number of strains of both species to determine the septation frequency in ascospores. In *M. majus* strains, the abundance of ascospores with three septa was higher than that of single-septum ascospores. Interestingly, ascospores with different numbers of septa are present in ascus; however, previous studies showed the same situation in *Sordariomycetes* [11,59].

The measurements of perithecia in the *M. seminicola* MFG 60138 strain did not show conspicuous differences from those in the other two species, and they were similar to the sizes indicated for maturing perithecia of this strain on OA: 110–149 μm [15].

Herein, we assessed the significant growth differences in *Microdochium* fungi between the species; large differences in growth were detected with respect to the temperature. *M. majus* and *M. nivale* had a relatively high growth rate at a wide range of temperatures, which determined the adaptability and the occurrence frequency of these fungi. All strains of *M. seminicola* grew at a considerably lower rate than the strains of other species.

*Microdochium* fungi are cited as a common cause of cereal pathogens in cooler regions [18,60,61,62]. This could be attributed to the dramatic inhibition of their growth at temperatures > 25 °C. It was speculated that *Microdochium* prefers low temperatures, as it is a psychrotolerant species. *M. nivale* from different locations in Slovakia grew better in vitro at temperatures < 20 °C [63]. However, Doohan et al. [60] showed that the isolates of *M. nivale* sensu lato of European origin grew optimally at 20–25 °C.

Furthermore, we tested the ability of strains of three *Microdochium* species to produce mycotoxins commonly found in filamentous fungi. Previous studies indicated that *M. nivale* did not produce trichothecenes [45,64,65,66] or zearalenone [67]. However, these studies examined strains that appeared to be *M. nivale* sensu lato. We did not detect any of the 31 analyzed secondary metabolites in the rice samples inoculated by *Microdochium* spp. strains.

The current study has provided further insights into the identity and prevalence of *Microdochium* fungi in cultivated cereals in Russia in diverse habitats. The results indicate that at least three *Microdochium* species occur on cereals. These species are morphologically similar but differ in geographical representation and environmental requirements. *M. seminicola* was predominant among *Microdochium* fungi in the West Siberian region of Russia. Our findings may contribute to the understanding of the large-scale patterns of fungal diversity in different ecosystems.

## Figures and Tables

**Table 1:** Origin of *Microdochium* and *Fusarium* strains used in the present work.

No.	MFG ^a^	Strain	Region ^b^	Origin	Year	Host	Tissue Origin
1	58968	*M. nivale*	CE	Bryansk	2012	wheat	grain
2	58972	*M. majus*	CE	Briansk	2012	wheat	grain
3	60127	*M. majus*	CE	Voronezh	2017	wheat	grain
4	60128	*M. majus*	CE	Voronezh	2017	wheat	grain
5	60136	*M. nivale*	CE	Voronezh	2017	wheat	grain
6	60210	*M. nivale*	CE	Voronezh	2017	wheat	grain
7	60214	*M. nivale*	CE	Belgorod	2018	wheat	snow mold
8	60216	*M. nivale*	CE	Belgorod	2018	wheat	snow mold
9	60217	*M. nivale*	CE	Belgorod	2018	wheat	snow mold
10	58962	*Microdochium* sp.	China		2016	oat	grain
11	58971	*M. majus*	NW	Kaliningrad	2012	wheat	grain
12	59022	*M. nivale*	NW	Leningrad	2016	oat	grain
13	59023	*M. majus*	NW	Leningrad	2016	wheat	grain
14	59028	*M. nivale*	NW	Pskov	2016	wheat	grain
15	59031	*M. nivale*	NW	Leningrad	2016	oat	grain
16	59100	*M. nivale*	NW	Leningrad	2016	wheat	grain
17	60231	*M. nivale*	NW	Leningrad	2012	rye	snow mold
18	58910	*M. nivale*	SE	Krasnodar	2016	wheat	spike
19	58912	*M. majus*	SE	Krasnodar	2016	wheat	spike
20	58926	*M. majus*	SE	Krasnodar	2016	wheat	leaf spot
21	58973	*M. majus*	SE	Stavropol	2014	wheat	grain
22	58976	*M. majus*	SE	Stavropol	2014	wheat	grain
23	59102	*M. majus*	SE	Krasnodar	2016	barley	grain
24	59103	*M. majus*	SE	Krasnodar	2016	barley	grain
25	59144	*M. nivale*	SE	Krasnodar	2016	wheat	grain
26	60016	*M. nivale*	SE	Krasnodar	2016	wheat	grain
27	60211	*M. nivale*	SE	North Ossetia	2017	wheat	grain
28	60227	*M. majus*	SE	Krasnodar	2001	barley	grain
29	60228	*M. nivale*	SE	Krasnodar	2001	barley	grain
30	60232	*M. nivale*	SE	Stavropol	2011	wheat	grain
31	60129	*M. majus*	WS	Novosibirsk	2017	wheat	grain
32	60131	*M. seminicola*	WS	Krasnoyarsk	2017	wheat	grain
33	60132	*M. seminicola*	WS	Krasnoyarsk	2017	wheat	grain
34	60138	*M. seminicola*	WS	Novosibirsk	2017	wheat	grain
35	60139	*M. seminicola*	WS	Tyumen	2017	wheat	grain
36	60140	*M. seminicola*	WS	Tyumen	2017	wheat	grain
37	60144	*M. nivale*	WS	Altay	2017	wheat	grain
38	58969	*M. nivale*	Volga	Mordovia	2012	wheat	grain
39	60116	*M. nivale*	Volga	Tatarstan	2017	wheat	grain
40	60117	*M. nivale*	Volga	Tatarstan	2017	wheat	grain
41	60118	*M. nivale*	Volga	Tatarstan	2017	wheat	grain
42	60119	*M. nivale*	Volga	Tatarstan	2017	wheat	grain
43	60123	*M. nivale*	Volga	Penza	2017	wheat	grain
44	60124	*M. nivale*	Volga	Penza	2017	wheat	grain
45	60125	*M. nivale*	Volga	Penza	2017	wheat	grain
46	60126	*M. nivale*	Volga	Penza	2017	wheat	grain
47	270611	*F. langsethiae*	Ural	Sverdlovsk	2018	oat	grain

^a^ MFG, the collection of the Laboratory of Mycology and Phytopathology in All-Russian Institute of Plant Protection; ^b^ the different regions of Russia: (NW) Northwestern, (CE) Central European, (SE) South European, and (WS) West Siberian.

**Table 2:** GenBank accession numbers of *Microdochium* spp. used in phylogenetic analysis.

Species	Strain	Country	Host	GenBank Accession Number			
				ITS	LSU	BTUB	RPB2
*Microdochium* sp.	MFG 58962	China	oat	**MN759664 ^a^**	**MN473989**	**MN817713**	**MN817743**
*M. seminicola*	MFG 60131	Western Siberia, Russia	wheat	**MN759667**	**MN473997**	**MN817716**	**MN817746**
*M. seminicola*	MFG 60132	Western Siberia, Russia	wheat	**MN759668**	**MN473998**	**MN817717**	**MN817747**
*M. seminicola*	MFG 60138	Western Siberia, Russia	wheat	**MN759669**	**MN473999**	**MN817718**	**MN817748**
*M. seminicola*	MFG 60139	Western Siberia, Russia	wheat	**MN759670**	**MN474000**	**MN817719**	**MN817749**
*M. seminicola*	MFG 60140	Western Siberia, Russia	wheat	**MN759671**	**MN474004**	**MN817720**	**MN817750**
*M. albescens*	CBS 290.79	Ivory Coast, West African	rice	KP859014	KP858950	KP859077	KP859123
*M. bolleyi*	CBS 540.92	Syria	barley	KP859010	KP858946	KP859073	KP859119
*M. colombiense*	CBS 624.94	Colombia	banana	KP858999	KP858935	KP859062	KP859108
*M. majus*	CBS 741.79	Germany	wheat	KP859001	KP858937	KP859064	KP859110
*M. neoqueenslandicum*	CBS 445.95	Netherlands	common rush	KP858997	KP858933	KP859060	KP859106
*M. nivale*	CBS 166205 T	United Kingdom	wheat	KP859008	KP858944	KP859071	KP859117
*M. phragmitis*	CBS 285.71 ET	Poland	*Phragmites australis*	KP859013	KP858949	KP859076	KP859122
*M. seminicola*	DAOM 250159	Switzerland	maize	KP859035	KP858971	KP859098	KP859144
*M. seminicola*	CBS 139951 T	Switzerland	maize	KP859038	KP858974	KP859101	KP8599147
*M. seminicola*	CBS 122707	Switzerland	maize	KP859011	KP858947	KP859074	KP859120
*M. trichocladiopsis*	CBS 623.77 T		wheat	KP858998	KP858934	KP859061	KP859107

^a^ Bold indicates the numbers of sequences obtained in this study.

**Table 3:** Sizes of the structures in mature ascomata of *Microdochium* strains.

*Microdochium* spp.	**Strain, MFG**	**Medium ^a^**	**Perithecial Structure**		1-Septum **Ascospores**		3-Septa **Ascospores**	
			Mean, μm	Range, μm	Mean, μm	Range, μm	Mean, μm	Range, μm
*M. majus*	60128	PSA	164 × 163	106-241 × 106-252	15.6 ×4.0	12.3-19.0 × 3.0-5.5	17.5 × 4.4	13.6-23.6 × 3.0-5.5
*M. majus*	60127	PSA	165 × 136	119-222 × 92-203	13.1 × 3.9	9.5-17.3 × 3.1-5.6	16.1 × 4.5	12.6-21.5 × 3.2-5.7
*M. majus*	60127	WS	153 × 123	141-171 × 100-152	14.4 × 4.6	10.6-18.2 × 3.5-6.0	16.3 × 5.1	12.3-19.6 × 4.2-6.2
*M. majus*	59102	PSA	154 × 119	118-193 × 95-117	15.7 ×4.3	12.8-18.0 × 3.5-5.1	17.3 × 4.3	13.0-21.8 × 3.5-5.1
*M. nivale*	60216	PSA	143 × 113	117-191 × 74-145	12.2 × 3.5	11.5-12.8 × 2.8-4.9	14.3 × 4.4	13.2-15.8 × 4.0-4.9
*M. seminicola*	60138	WS	128 × 139	101-168 × 90-100	–	–	17.6 × 4.0	16.5-18.2 × 3.5-4.5

^a^ PSA, potato sucrose agar; WA, wheat stem.

## Supplementary Materials

The following are available online at https://www.mdpi.com/2076-2607/8/3/340/s1, Figure S1: (A) Species distribution of *Microdochium* strains collected in the different geographic regions of Russia; (B) The length and width of three-septate conidia in *Microdochium* fungi. Figure S2: The symptoms of diseases of winter wheat, caused by *Microdochium* fungi: (A) *Microdochium* head blights; (B) seed-born *Microdochium* fungi; (C) necrotic lesions on the flag leaves; (D) ascomata on leaf sheaths near the base of the stem; (E) mature perithecium with revealed solitary ascospores (scale bar = 50 µm); and (F,G) snow mold in the field.
