# Supplementary figures and images for: Evidence of Microdochium Fungi Associated with Cereal Grains in Russia
Source: Microorganisms. 2020 Feb 28;8(3):340. doi: 10.3390/microorganisms8030340 (PMC7143527; doi:10.3390/microorganisms8030340)

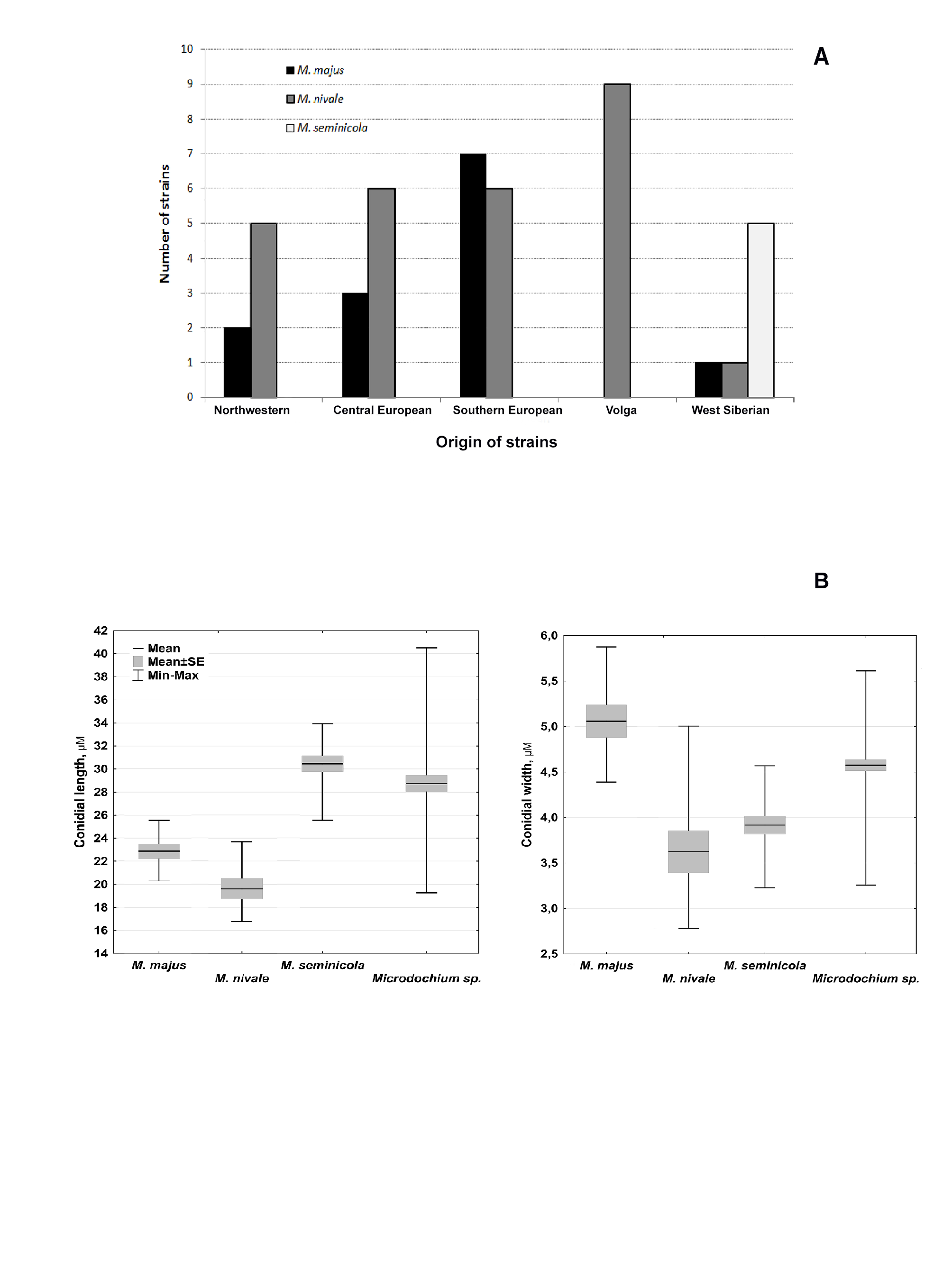

Supplement: Supplementary file 1 [file microorganisms-08-00340-s001.zip › microorganisms-722694-supplementary S1.tif]

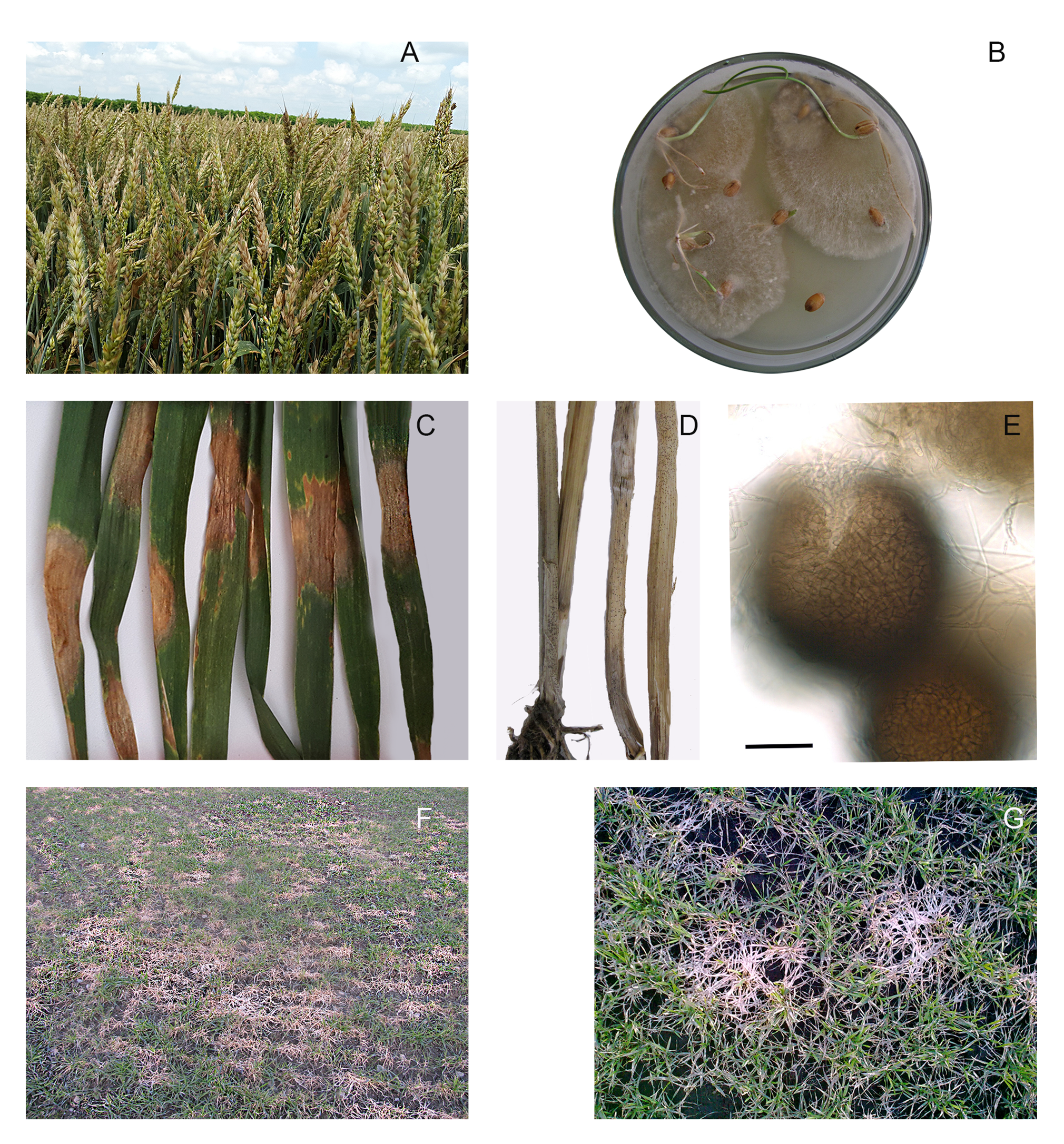

Supplement: Supplementary file 1 [file microorganisms-08-00340-s001.zip › microorganisms-722694-supplementary S2.tif]
